# Clinical Study on Causative Factors and Recurrence of Choledocholithiasis

**DOI:** 10.1155/DTE.3.81

**Published:** 1996

**Authors:** Hajime Hoshi, Yoshihiro Sakai

**Affiliations:** Third Department of Internal Medicine Ohashi Hospital Toho University School of Medicine 2-17-6 Ohashi, Meguro-ku Tokyo 153 Japan

## Abstract

To identify factors involved in choledocholithiasis, clinical characteristics were studied using univariate and multivariate analyses. Factors involved in recurrence were also investigated. The subjects consisted of 51 patients with calcium bilirubinate stones (B group) and 52 patients with cholesterol stones (C group). All patients had choledocholithiasis and underwent lithotripsy by endoscopic sphincterotomy (EST) during the past 9 years. Twenty variables, including clinical symptoms and endoscopic retrograde cholangiopancreatography (ERCP) findings, were analyzed using a Statistical Analysis System (SAS) software package. Univariate analysis were done using Student's t-test and the chi-square test. Multivariate analyses were done by stepwise logistic regression analysis. In univariate analyses, there were significant differences between the B group and C group in nine variables: age, common bile duct diameter, common hepatic duct diameter, common bile duct stone diameter, cystic duct diameter, and the presence of gallbladder stones, atypical arrangement of the hepatic duct, parapapillary diverticulum, and large parapapillary diverticulum. In multivariate analysis, the four variables of no gallbladder stone, large parapapillary diverticulum, cystic duct less than 8 mm, and atypical arrangement of the hepatic duct were significant independent factors for the development of stones in the B group, with relative risks of 37.75, 16.73, 5.56, and 5.49, respectively. The results indicated that calcium bilirubinate stones were frequently associated with parapapillary diverticulum and abnormal arrangement of the bile duct. The formation of these stones was attributed to chronic biliary stasis caused by dysfunction of the biliary tract, including the papilla. In contrast, most cholesterol stones found in the common bile duct had apparently descended from the gallbladder. Common bile duct stones recurred after EST in 9 patients, all of whom had calcium bilirubinate stones. On ERCP, recurrence was found to be frequently associated with gallbladder stones, large parapapillary diverticula, and atypical arrangement of the hepatic duct. Patients with these characteristics on initial ERCP should therefore receive appropriate treatment and undergo strict follow-up observations owing to the increased risk of recurrence caused by dysfunction of the biliary tract.


*Diagnostic and Therapeutic Endoscopy*, 1996, Vol. 3, pp. 81–91
Reprints available directly from the publisher
Photocopying permitted by license only

© 1996 OPA (Overseas Publishers Association)
Amsterdam B.V. Published in The Netherlands
by Harwood Academic Publishers
Printed in Malaysia

### Clinical Study on Causative Factors and Recurrence of Choledocholithiasis

HAJIME HOSHI* and YOSHIHIRO SAKAI

*Third Department of Internal Medicine, Ohashi Hospital, Toho University School of Medicine, 2-17-6 Ohashi, Meguro-ku, Tokyo 153, Japan*

*(Received 8 November 1995; In final form 1 June 1996)*

**To identify factors involved in choledocholithiasis, clinical characteristics were studied using univariate and multivariate analyses. Factors involved in recurrence were also investigated. The subjects consisted of 51 patients with calcium bilirubinate stones (B group) and 52 patients with cholesterol stones (C group). All patients had choledocholithiasis and underwent lithotripsy by endoscopic sphincterotomy (EST) during the past 9 years. Twenty variables, including clinical symptoms and endoscopic retrograde cholangiopancreatography (ERCP) findings, were analyzed using a Statistical Analysis System (SAS) software package. Univariate analysis were done using Student's t-test and the chi-square test. Multivariate analyses were done by stepwise logistic regression analysis. In univariate analyses, there were significant differences between the B group and C group in nine variables: age, common bile duct diameter, common hepatic duct diameter, common bile duct stone diameter, cystic duct diameter, and the presence of gallbladder stones, atypical arrangement of the hepatic duct, parapapillary diverticulum, and large parapapillary diverticulum. In multivariate analysis, the four variables of no gallbladder stone, large parapapillary diverticulum, cystic duct less than 8 mm, and atypical arrangement of the hepatic duct were significant independent factors for the development of stones in the B group, with relative risks of 37.75, 16.73, 5.56, and 5.49, respectively. The results indicated that calcium bilirubinate stones were frequently associated with parapapillary diverticulum and abnormal arrangement of the bile duct. The formation of these stones was attributed to chronic biliary stasis caused by dysfunction of the biliary tract, including the papilla. In contrast, most cholesterol stones found in the common bile duct had apparently descended from the gallbladder. Common bile duct stones recurred after EST in 9 patients, all of whom had calcium bilirubinate stones. On ERCP, recurrence was found to be frequently associated with gallbladder stones, large parapapillary diverticula, and atypical arrangement of the hepatic duct. Patients with these characteristics on initial ERCP should therefore receive appropriate treatment and undergo strict follow-up observations owing to the increased risk of recurrence caused by dysfunction of the biliary tract.**

*Keywords:* Choledocholithiasis, endoscopic retrograde cholangiopancreatography, recurrent stones

*Corresponding author. Tel.: 03-3468-1251. Fax: 03-3468-1269.

### I. INTRODUCTION

There have been many basic and clinical studies on cholecystolithiasis. A better understanding of the factors responsible for cholecystolithiasis has contributed to more accurate diagnosis and to improved therapeutic techniques [1–5]. However, relatively few studies have focussed on the etiology and diagnosis of choledocholithiasis, and the clinical characteristics of this disease are still poorly understood. The authors therefore studied the factors involved in the genesis of choledocholithiasis by univariate and multivariate analyses in patients undergoing endoscopic sphincterotomy (EST). These factors included clinical signs and symptoms of patients with choledocholithiasis who underwent transpapillary lithotomy, cholangiographic findings on endoscopic retrograde cholangiopancreatography (ERCP), and the configuration of the duodenal papilla. In addition, the characteristics of the bile duct and causative factors were studied in patients with recurrent stones.

### II. SUBJECTS AND METHODS

### 1. Subjects

During the 9-year period between January 1986 and December 1994, 159 patients with choledocholithiasis underwent stone removal by EST. Sixty-two of these patients had stones of the common bile duct alone, and 97 had stones of the gallbladder and the common bile duct. Seventy of the patients were men, and 89 were women. Their mean age was 69.6 ± 12.9 years. Mechanical lithotripsy was performed using a lithotripter in 53 patients. Lithotripsy using a basket and balloon catheter was performed in 90 patients. Electrohydraulic lithotripsy (EHL) under peroral cholangioscopy was performed in 3 patients. Transpapillary lithotomy could not be performed in 10 patients, who instead underwent percutaneous transhepatic cholangioscopic lithotripsy (PTCSL). The stones were spontaneously discharged in three patients (Table I). One hundred fifteen patients in whom the stones were able to be retrieved (excluding spontaneous discharge and retrieval of only minute quantities of stone) and who had cholangiograms that permitted interpretation and measurement of the biliary tract were studied. Furthermore, patients undergoing cholecystectomy or gastrectomy, which may alter the diameter of the bile duct, and patients who concurrently had intrahepatic stones, which are considered to be caused by factors different from those producing stones of the common bile duct, were also excluded.

TABLE I Characteristics of patients with choledocholithiasis who underwent endoscopic sphincterotomy (EST)

| | | 1986, 1 ~ 1994, 12 |
|---|---|---|
| CBD stone | | 159 |
| CBD alone | | 62 |
| With GB stone | | 97 |
| Lithotripsy | | |
| Mechanical lithotripsy | | 53 |
| Basket & baloon | | 90 |
| EHL | | 3 |
| PTCSL | | 10 |
| Spontaneous discharge | | 3 |
| M:F | 70:89 | |
| Mean age | 69.6 ± 12.9 | |

CBD: common bile duct
GB: gallbladder
EHL: electrohydraulic lithotripsy
PTCSL: percutaneus transhepatic cholangioscopic lithotripsy

### 2. Stone Analysis and Analytical Methods

The components of common bile duct stones were analyzed by infrared (IR) spectroscopy. Fifty-one patients had calcium bilirubinate stones (B group), and 52 had cholesterol stones (C group). In addition, nine patients had mixed stones containing at least 30% calcium bilirubinate and cholesterol (mixed stone group), and five patients had black stones (black stone group), evaluated by appearance of the surface and cut sections of the stone (Table II).

Differences between the B group and C group were studied by univariate analysis of the following clinical symptoms and laboratory findings at admission: fever (≥37°C), abdominal pain (epigastric or right hypochondrial spontaneous pain or tenderness), jaundice (serum total bilirubin ≥3.0 mg/dl), elevated levels of biliary enzymes (serum alkaline phosphatase [A1P] or serum leucine aminopeptidase [LAP]), and the presence of

TABLE II Type of common bile duct stones

| | No. of patients |
|---|---|
| Calcium bilirubinate stone (B group) | 51 |
| Cholesterol stone (C group) | 52 |
| Mixed stone | 9 |
| Black stone | 5 |
| | 117 |

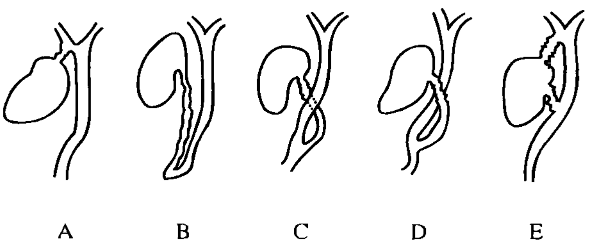

FIGURE 1 Classification of abnormal arrangements of the cystic duct cholangitis (abdominal pain + elevated biliary enzymes + leukocyte count ≥10,000 or C-reactive protein [CRP] positive) or cholecystitis (enlargement of the gallbladder and thickening of its wall on abdominal ultrasonography + leukocyte count ≥10,000 or CRP positive).

B group and C group were similarly compared by univariate analysis for variables determined in cholangiograms on ERCP and endoscopic findings of the duodenal major papilla (the presence or absence of gallbladder stones, gallbladder stone diameter, the diameter and number of common bile duct stones, common bile duct diameter, common hepatic duct diameter, cystic duct diameter, the presence or absence of bile duct stenosis, the presence or absence of an atypical arrangement of the cystic duct, the structure of the hepatic duct confluence, and the presence or absence of a parapapillary diverticulum and a large parapapillary diverticulum). For cholangiograms, films that included the intrahepatic duct and gallbladder in the initial ERCP images were selected, in principle. If the initial images were unsatisfactory, the cholangiograms taken after EST treatment were used. The images were interpreted and measured by several specialists, and the maximum diameter was measured for each specified site. Abnormal arrangement of the cystic duct was classified according to the questionnaire survey used by the 22nd Meeting of the Japanese Study Group on Biliary Surgery (Fig. 1) [6]. The arrangement of the hepatic duct was classified as typical, in which all intrahepatic ducts join the left and right main trunks of the hepatic duct before forming the common bile duct, or atypical, in which the left, right or both intrahepatic ducts join the common bile duct without forming the main hepatic duct (Fig. 2). The parapapillary diverticulum was examined endoscopically, and a large parapapillary diverticulum was considered to be present when the opening of the diverticulum was two or more times the transverse diameter of the papilla, or if the site in contact with the main papilla was a half circumference or more (Fig. 3). Statistical analysis was done using a Statistical Analysis System (SAS) software package. The significance of the results of univariate analysis was tested using Student's t-test and the chi-square test. $p$ values less than 0.05 were considered to indicate statistical significance. Multivariate analysis was done by stepwise logistic regression analysis of the variables used in univariate analysis.

After performing EST, complete lithotripsy was confirmed by ERCP images or by peroral cholangioscopy. The subsequent recurrence of common bile duct stones was studied in 102 patients who were able to be observed for 2 years or more at the outpatient clinic of this hospital or affiliated hospitals. Recurrence was defined as the confirmation of a stone on ERCP more than 1 year after removal of the initial stone. Patients in whom a stone could not be confirmed on cholangioscopy were not considered to have had recurrence, even when papillary enlargement or purulent bile were present.

### III. RESULTS

### 1. Comparison Between B Group and C Group by Univariate Analysis

#### *(1) Clinical Symptoms and Findings at Admission*

Symptoms other than abdominal pain were noted in less than half of the patients in each group. Seven patients (14%) in the B group and 10 (19%) in the C

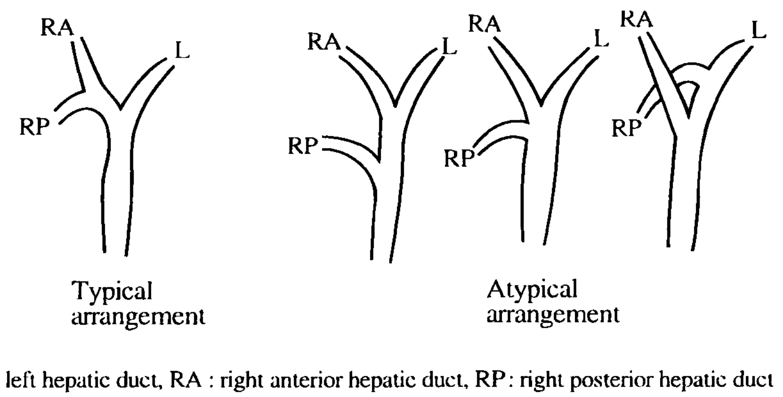

FIGURE 2 Typical and atypical arrangements of the hepatic duct.

group were asymptomatic (combined total, 17 patients, 17%).

Elevation of biliary enzymes was seen in most patients in both groups, and 24 patients (47%) in the B group and 20 (38%) in the C group (combined total, 44 patients, 43%) presented with signs and symptoms of cholangitis. Among these patients, only one in the B group and two in the C group had acute obstructive suppurative cholangitis (AOSC), which was associated with shock and disturbed consciousness. Both of these patients required emergency EST and drainage. Cholecystitis was concurrently present in 8 patients (16%) in the B group and 11 patients (21%) in the C group (combined total, 19 patients, 18%). There was no significant difference between the groups in any of these characteristics (Table III).

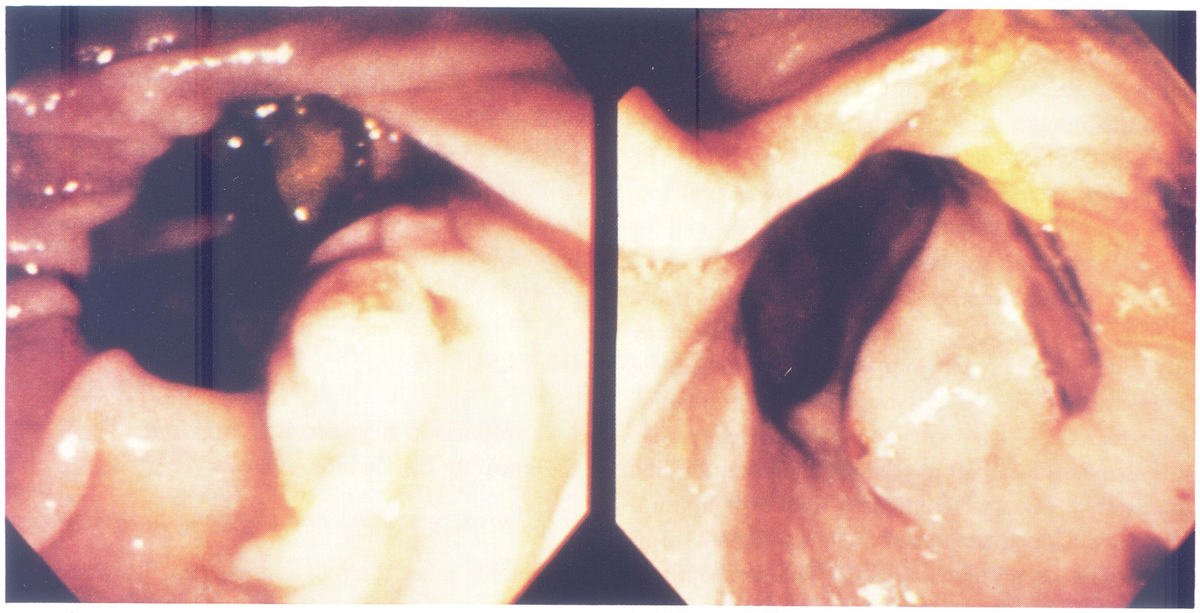

FIGURE 3 Endoscopic findings of a large parapapillary diverticulum

TABLE III Clinical signs and symptoms at admission

| Characteristic | B group (n = 51) | C group (n = 52) | *p* value |
|---|---|---|---|
| Fever ↑ + | 24 | 16 | |
| – | 27 | 36 | 0.090 |
| Abdominal pain + | 40 | 39 | |
| – | 11 | 13 | 0.680 |
| Jaundice + | 22 | 21 | |
| – | 29 | 31 | 0.777 |
| Symptoms – | 7 | 10 | |
| + | 44 | 42 | 0.452 |
| Bile duct enzymes ↑ | 42 | 43 | |
| → | 9 | 9 | 0.964 |
| Cholangitis + | 24 (1) | 20 (2) | |
| – | 27 | 32 | 0.378 |
| Cholecystitis + | 8 | 11 | |
| – | 43 | 41 | 0.474 |

( ): Acute obstructive suppurative colangitis $\chi^2$-test
B group: Calcium bilirubinate stones
C group: Cholesterol stones

### *2) Age and Maximum Diameters at Each Site in ERCP Cholangiograms*

The mean age of the patients was significantly higher in the B group than in the C group ($p < 0.001$). The diameters of the common bile duct and the common hepatic duct were also significantly greater in the B group ($p < 0.001$, P = 0.005). Similarly, the diameter of common bile duct stones was significantly greater in the B group than in the C group ($p < 0.001$). In contrast, the diameter of the cystic duct was significantly greater in the C group than in the B group ($p < 0.001$) (Table IV).

### *3) ERCP Cholangiograms and Endoscopic Findings*

Gallbladder stones were concurrently present in significantly more patients in the C group than the B group ($p < 0.001$). The arrangement of the hepatic duct was atypical in a significantly higher proportion of patients in the B group ($p = 0.004$). A parapapillary diverticulum was present in significantly more patients in the B group ($p < 0.001$), and large parapapillary diverticulum were more remarkable in the B group ($p < 0.001$). There was no difference between the groups with respect to the number of common bile

TABLE IV Age of the patients, biliary tract diameter, and stone diameter.

| Characteristic | B group (n = 51) | C group (n = 52) | *p* value |
|---|---|---|---|
| Age | 74.7 ± 8.3 | 63.9 ± 14.8 | <0.001 |
| Common bile duct | 17.6 ± 4.1 | 14.7 ± 4.0 | <0.001 |
| Common hepatic duct | 14.9 ± 3.7 | 12.8 ± 3.5 | 0.005 |
| Cystic duct | 6.7 ± 2.2 | 8.8 ± 2.9 | <0.001 |
| CBD stone | 15.1 ± 7.6 | 10.4 ± 4.2 | <0.001 |
| GB stone | 13.7 ± 6.9 | 13.5 ± 6.9 | 0.884 |

Mean ± SD (mm) Student's-test duct stones, the presence of common bile duct stenosis, and an abnormal arrangement of the cystic duct (Table V).

### 2. Comparison Between B Group and C Group by Multivariate Analysis

The eight variables selected for stepwise analysis (concurrent presence of gallbladder stones, hepatic duct arrangement, parapapillary diverticulum, large parapapillary diverticulum, age, common bile duct stone diameter, common bile duct diameter, and cystic duct diameter) underwent multivariate analysis using a multiple logistic model. Since the numerical variables of age, common bile duct stone diameter,

TABLE V ERCP and endoscopic findings

| Characteristic | B group (n = 51) | C group (n = 52) | *p* value |
|---|---|---|---|
| GB stone + | 26 | 50 | |
| – | 25 | 2 | <0.001 |
| CBD solitary stone | 21 | 23 | |
| multiple stones | 30 | 29 | 0.754 |
| CBD stenosis + | 15 | 10 | |
| – | 36 | 42 | 0.228 |
| HD arrangement, atypical | 34 | 20 | |
| typical | 17 | 32 | 0.004 |
| CD arrangement, abnormal | 21 | 18 | |
| normal | 30 | 34 | 0.429 |
| PD + | 42 | 9 | |
| – | 9 | 43 | <0.001 |
| Large PD + | 29 | 2 | |
| – | 22 | 50 | <0.001 |

HD: Hepatic bile duct.
CD: Cystic duct. $\chi^2$-test
PD: Parapapillary diverticulum.

common bile duct diameter, and cystic duct diameter do not have a normal distribution, they were expressed as medians. The four variables of gallbladder stones ($p = 0.00166$), large parapapillary duodenal diverticulum ($p = 0.01520$), hepatic duct arrangement ($p = 0.03534$), and cystic duct diameter ($p = 0.03799$) were found to be significant. The relative risks of these variables as independent factors in the B group were as follows: no gallbladder stone, 37.75; large parapapillary diverticulum, 16.73; cystic duct diameter less than 8 mm, 5.56, and atypical hepatic duct arrangement 5.49 (Table VI-a, b).

### 3. Patients with Recurrence of Common Bile Duct Stones

Stones recurred in 9 patients (4 men and 5 women). These patients were elderly (mean age, 77.7 ± 9.2 years). The mean time to recurrence was 29 months, and most cases of recurrence occurred after about 2 years. Seven of these patients had gallbladder stones, including 6 cases with medium to large stones measuring over 15 mm in diameter. It was thus unlikely that the stones had descended from the gallbladder to the common bile duct. One patient had multiple stones each less than 10 mm in diameter; recurrence occurred repeatedly over a short period. The initial stones were treated by basket lithotripsy in 4 patients, and by mechanical basket lithotripsy in 5 patients. Complete lithotripsy was confirmed cholangiographically by postoperative balloon ERC. Most of these patients had early-stage disease, and the remaining stones were confirmed by peroral cholangioscopy in only 2 of the 9 cases. Virtually all recurrent stones were more than 15 mm in diameter. Mechanical basket lithotripsy was performed in all patients who had a recurrence. The stones were completely removed in 7 patients. One patient died of septicemia provoked by AOSC, and another died of acute left heart failure during lithotripsy. All recurrent cases had calcium bilirubinate stones. Of the 9 patients with recurrence, only 4 had an abnormal arrangement of the cystic duct, whereas 7 had an atypical arrangement of the hepatic duct, and 8 had large parapapillary duodenal diverticulum. EST at the time of recurrence required a small incision up to the covering fold in 5 patients, a medium incision up to the middle of the oral protrusion in 3 patients, and a large incision reaching the oral protrusion in 1 patient (Table VII).

TABLE VI-a Factors contributing to CBD stones according to multivariate analysis with a logistic regression model

| Variable | Estimate | Standard error | Standard deviation | $p$ value |
|---|---|---|---|---|
| GB stone (+ → 1, – → 0) | –3.63098 | 1.12072 | –3.23986 | 0.00166 |
| HD arrangement (atypical → 1, typical → 0) | –1.70369 | 0.79774 | –2.13563 | 0.03534 |
| PD (+ → 1, – → 0) | –1.25149 | 0.93670 | –1.33607 | 0.18479 |
| Large PD (+ → 1, – → 0) | –2.81720 | 1.13901 | –2.47338 | 0.01520 |
| Age (73 ≦ → 1, 73 > → 0) | –0.82873 | 0.74576 | –1.11126 | 0.26932 |
| CBD stone (12 mm ≦ → 1, 12 mm > → 0) | –0.01723 | 0.85742 | –0.02010 | 0.98399 |
| CBD (16 mm ≦ →1, 16 mm > → 0) | –1.14305 | 0.81648 | –1.39998 | 0.16485 |
| Cystic duct (8 mm ≦ →1, 8 mm >→ 0) | 1.71639 | 0.81538 | 2.10502 | 0.03799 |

GB: gallbladder, HD: hepatic bile duct, PD: Parapapillary diverticulum, CBD: common bile duct

TABLE VI-b Relative risks of significant variables in the B group by a logistic regression model

| Variables | Risk ratio | 95% Confidence limits | $p$ value |
|---|---|---|---|
| GB stone – | 37.75 | 4.20 ~ 339.54 | 0.00166 |
| Large PD + | 16.73 | 1.79 ~ 155.97 | 0.01520 |
| Cystic duct (8 mm >) | 5.56 | 1.13 ~ 27.51 | 0.03799 |
| HD atypical arrangement | 5.49 | 1.15 ~ 26.24 | 0.03534 |

Logistic regression procedure

TABLE VII Patients with recurrence of CBD stones

| Case | 1 | 2 | 3 | 4 | 5 | 6 | 7 | 8 | 9 |
|---|---|---|---|---|---|---|---|---|---|
| Sex | F | F | M | M | F | M | M | M | F |
| Age | 86 | 77 | 85 | 78 | 64 | 80 | 81 | 90 | 58 |
| GB stone | – | + | + | + | + | + | – | + | + |
| GBS size (mm) | | 30 | 28 | 15 | 10 | 19 | | 15 | 18 |
| CBDS size (mm) | 22 | 12 | 10 | 22 | 8 | 14 | 20 | 27 | 15 |
| r-CBDS size (mm) | 20 | 17 | 13 | 20 | 18 | 15 | 18 | 20 | 17 |
| Analysis | Bil | Bil | Bil | Bil | Bil | Bil | Bil | Bil | Bil |
| Period (m) | 23 | 44 | 25 | 57 | 24 | 24 | 20 | 28 | 15 |
| Lithotripsy | Lt | Bt | Bt | Lt | Bt | Bt | Lt | Lt | Lt |
| r-lithotripsy | Lt | Lt | Lt | Lt | Lt | Lt | Lt | Lt | Lt |
| EST size | M | M | S | S | M | S | S | L | S |
| CD abnormal arrangement | – | – | + | + | – | + | + | – | – |
| HD atypical arrangement | + | + | + | + | + | + | – | + | – |
| Large PD | – | + | + | + | + | + | + | + | + |
| Clinical course | A | A | A | A | A | A | D | D | A |

r-CBDS: recurrent common bile duct stone (mm), Bil: calcium bilirubinate stone, period: period to recurrence, r-lithotripsy: lithotripsy at recurrence, Lt: lithotripter, Bt: basket L: large, M: medium, S: small, A: alive, D: died

### IV. DISCUSSION

Choledocholithiasis is frequently accompanied by symptoms of obstructive jaundice and cholangitis, but some cases are asymptomatic, which often precludes early diagnosis. As this disease occurs primarily in elderly patients, symptoms may be vague. In fact, in this study about 17% of the patients were asymptomatic, and two-thirds of these patients had normal biliary enzyme levels. On the other hand, cholangitis accompanied by symptoms, signs of inflammation, and elevated levels of biliary enzymes was noted in more than 40% of the patients. Although rare, once AOSC develops it grows progressively more serious and requires emergency drainage. Early diagnosis is therefore vital, and treatment is essential even in asymptomatic patients.

In the field of internal medicine, transpapillary lithotomy by EST is generally employed to treat choledocholithiasis, and we have performed EST as the procedure of choice since 1985 [7,8]. Cases complicated by confluence stones and intrahepatic stones are treated by PTCSL, which has also produced good results [9,10]. By selecting between these two routes for endoscopic treatment, most cases of choledocholithiasis can be managed effectively. In the field of surgery, choledochotomy is assigned a high priority, and the indications for transpapillary surgery differ depending on the institution. Internists and surgeons thus still differ in their approaches to treatment [11,12,13]. Recently, extracorporeal shock wave lithotripsy (ESWL) has also been used to treat bile duct stones [14]. In the future, more refined techniques for diagnosis and a corresponding wider assortment of treatment options will most likely become available. Therefore, assessment of differences between bilirubinate stones and cholesterol stones based on ERCP images and clinical symptoms is considered to be useful for diagnosis and the selection of the optimal procedure for treatment.

Stones were classified according to the classification proposed by the proposal of the Gallstone Study Group of the Japanese Society of Gastro-Enterology [15], but black stones were classified separately because their components have not been fully defined and because IR analysis is still inadequate [16]. Pure cholesterol stones and mixed-component stones may be grouped together under the general category of cholesterol stones, but it is often difficult to distinguish mixed stones from bilirubinate stones [16]. For analysis, we therefore created a separate category for mixed stones that could not be clearly allocated to B group or C group.

Bilirubinate stones were formerly the most prevalent type of stone in Japan. Their formation has been widely accepted to involve the bacterial β-glucuronidase theory, and the mechanism for stone biosynthesis has been demonstrated in vitro [17,18]. Formerly, bilirubinate stones were found predominantly in elderly patients and accounted for the majority of common bile duct stones. Recently, however, there has been a trend toward a relative decrease in bilirubinate stones [19], and in this study there were comparable numbers of patients with bilirubinate stones (51) and those with cholesterol stones (52). Furthermore, although concurrent gallbladder stones were not uncommon in the B group, over half the patients in this group had common bile duct stones alone. In contrast, most patients in the C group concurrently had gallbladder stones, and cases of solitary common bile duct stones were extremely rare. Therefore, the high number of cholesterol stones in the common bile duct was attributed to gallbladder stones descending to the common bile duct rather than a high rate of biosynthesis of cholesterol stones inside the common bile duct.

The significantly greater diameter of common bile duct stones in the B group than in the C group was considered to reflect the gradual formation of bilirubinate stones within the bile duct; these stones only rarely descend from the gallbladder. The significantly greater caliber of the bile duct in the B group was apparently caused by prolonged elevation of pressure within the bile duct and biliary stasis, but the effects of age-related changes and papillary dysfunction also merit consideration. In contrast, the smaller stone and common bile duct diameters and the significantly greater cystic duct diameter in the C group, revealed by univariate analysis, and the fact that the cystic duct diameter was related to stone formation in the C group, as demonstrated by multivariate analysis, indicate that stones in this group descended from the gallbladder and passed through the cystic duct to the common bile duct.

Since the description of the "Papillensyndrom" by Lemmel [20], parapapillary diverticulum have been considered to be associated with cholelithiasis, and a particularly strong relation with common bile duct stones has been suggested [21,22,23]. Many reports on the mechanism of stone formation have claimed that mechanical retraction of the end of the common bile duct [20,21] or Oddi's muscle, or papillary dysfunction caused by continuous physical stimulation [22,23] elevate bile duct pressure, promote biliary stasis, and disturb biliary flow, but the details are still unclear owing to the lack of adequate basic research. Kimura *et al.* [24] reported no association between pathological evidence of parapapillary duodenal diverticula pathologically and histological changes of the papillary region. Clinically, however, diverticula were considered to be involved in stone formation. In addition, although most diverticula have been found on the oral side of the papilla, there have been few studies on diverticulum size [23,25], and measurement methods have differed depending on the institution. We have designated a large parapapillary diverticulum to be present when the opening of the diverticulum was two or more times the transverse diameter of the main duodenal papilla, or if the site in contact with the main papilla was a half circumference or more. In multivariate analyses, a large papillary diverticulum was the second most strongly associated factor with stone formation in the B group, following no gallbladder stone. With regard to the relation between parapapillary diverticulum and stone recurrence, Fuji *et al.* [26] reported that parapapillary duodenal diverticula were seen in all patients who had recurrent stones of the common bile duct, but there was no mention of size. In the present study, the presence of large diverticulum in the majority of patients with recurrence clearly indicates long-term functional or organic disturbances of the lower biliary tract, including the papillary region. These patients were therefore at greater risk for the development of severe biliary stasis and infection.

The biliary tract is known to show a diverse variety of arrangements but a concept defining abnormal arrangement has yet to be established [27]. Recently, Kuji *et al.* [6] classified abnormal arrangement of the cystic duct based on the results of a questionnaire survey of members of the Japanese Study Group for Biliary Surgery (Fig. 1). The results of this survey indicated the incidence of abnormal patterns of the cystic duct to be about 5%, which is distinctly lower than in North America and Europe [28,29]. We previously reported on abnormal arrangement of the cystic duct in patients with cholelithiasis [30], and in cholecystolithiasis the incidence an abnormal arrangement of the cystic duct was relatively high (13%). In the present study, there was a remarkably high rate of abnormal arrangement of the cystic duct, found in 39 (38%) of the 103 patients with common bile duct stones in the B group and C groups. This suggests that abnormal arrangement of the cystic duct is associated with some dysfunction of the cystic duct, which may be involved in not only the formation of gallbladder stones but also stones descending from the gallbladder. Many reports have described abnormal configurations of the bile ducts, particularly the hepatic duct [27,31]. However, in Japan there are considerable differences among institutions concerning the nomenclature for the accessory hepatic ducts, and no standardized classification is available. We therefore considered a typical arrangement to be present when the intrahepatic bile ducts united in one left and one right main trunk, and all other arrangements were defined as atypical. In other words, these latter cases were classified as atypical arrangement of the hepatic duct. There has been no study of the association between the arrangement of the hepatic duct and function of the papillary region. In our series, an atypical arrangement of the hepatic duct was present in 54 (52%) of 103 cases of common bile duct stones. In a series of 318 cases of gallbladder stones diagnosed during the same period as the present study, an atypical arrangement of the hepatic duct was found in only 87 patients (27%). The incidence of atypical hepatic duct arrangement was thus distinctly higher in patients with common bile duct stones. Moreover, multivariate analysis indicated that an atypical arrangement was associated with stone formation in the B group. This suggested that an atypical arrangement of the hepatic duct was involved in some type of dysfunction of the bile ducts as a whole, including the papillary region.

EST and other types of endoscopic therapy for common bile duct stones have been demonstrated to produce excellent therapeutic results [32,33]. Recently, techniques such as mother-baby-type peroral cholangioscopy and biliary drainage have been routinely used for the diagnosis and treatment of cholelithiasis and other biliary tract diseases. Particularly remarkable progress has been made with ERCP and its applied techniques [7,8]. On the other hand, as more long-term follow-up results of EST therapy become available, late-stage complications, such as stone recurrence and late-stage cholangitis, have emerged as problems [32–34]. Many previous reports have assessed the relation with recurrence rate or concurrent gallbladder stones. As stated by Ikeda *et al.* [32], it is inappropriate to evaluate recurrence by merely surveying a series of patients at a given point in time, and late-stage cholangitis may often occur in patients in whom gallbladder stones are left untreated [32,34]. We studied stone recurrence based principally on ERCP images. Among the patients with recurrence, all had bilirubinate stones and a particularly high proportion had an atypical arrangement of the hepatic duct or large parapapillary duodenal diverticula. Furthermore, the high number of recurrent cases with gallbladder stones implies that the risk of recurrence must be considered in the B group (in patients with bilirubinate stones), which has a risk of late-stage cholecystitis as well as concurrent papillary dysfunction in case that gallbladder stones are allowed to remain. Since many patients with common bile duct stones are elderly and have underlying diseases, stone recurrence may trigger serious complications. In the present study, two patients died during lithotripsy; follow-up is therefore extremely important. These findings indicate that patients found at the time of initial treatment to have large parapapillary diverticula, an abnormal arrangement of the hepatic duct, and gallbladder stones have an increased risk of recurrence. Gallbladder stones in such patients should therefore be treated by cholecystectomy or other suitable procedures. All patients in whom gallbladder stones are left untreated require strict follow-up.

### V. CONCLUSION

Clinical symptoms, ERCP images, and endoscopic findings of the duodenal papillary region in patients with choledocholithiasis who endoscopically underwent lithotripsy by EST were studied using univariate and multivariate analysis to identify factors involved in the formation of stones. The following conclusions were obtained:

1. As clinical symptoms and findings, symptoms of acute cholangitis were noted in 24 patients (47%) in the B group and 20 patients (38%) in the C group. Although AOSC developed in only one patient in the B group, and two in the C group, all of these patients required emergency EST. Overall, about 17% of the patients were asymptomatic.
2. The age of the patients was significantly higher in the B group than in the C group. Common bile duct diameter, common hepatic duct diameter, and common bile duct stone diameter were significantly greater in the B group than in the C group. In contrast, cystic duct diameter was significantly greater in the C group.
3. In cholangiograms on ERCP and endoscopic images, the absence of gallbladder stones, atypical arrangement of the hepatic duct, parapapillary duodenal diverticula, and large parapapillary diverticula were significantly more frequent in the B group. There was no difference between the groups in the number of common bile duct stones or the presence of common bile duct stenosis and abnormal arrangement of the cystic duct.
4. In multivariate analysis, the four variables of no gallbladder stone, large parapapillary duodenal diverticulum, cystic duct less than 8 mm, and atypical arrangement of the hepatic duct were significant independent factors for the development of stones in the B group, with relative risks of 37.75, 16.73, 5.56, and 5.49, respectively.
5. Common bile duct stones recurred in 9 patients. The mean period to relapse was 29 months. All cases of recurrent stones were treated by mechanical basket lithotripsy. Complete lithotripsy was possible in seven patients, and the other two patients died. All recurrent cases had bilirubinate stones and the majority were associated with gallbladder stones, atypical arrangement of the hepatic duct, and large parapapillary duodenal diverticula.

### *References*

[1] Carey, M. C. and Cahalane, M. J. (1988). Whither biliary sludge? *Gastroenterol.*, **95**, 508–523.

[2] Burnstein, M. J., Ilson, R. G., Petrunka, C. N. *et al.* (1983). Evidence for a potent nucleating factor in the gallbladder bile of patients with cholesterol gallstones. *Gastroenterol.*, **85**, 801–807.

[3] Isa, T. and Muto, Y. (1991). Morphological changes of the gallbladder and gallstone formation. *J. Bil. Tract and Panc.*, **12**, 963–968 (in Japanese).

[4] Micami, S., Tsuchiya, Y., Natsuki, Y. *et al.* (1991). Ultrasonographic Imaging of the types of gallstones. *J. Bil. Tract and Panc.*, **12**, 1199–1204 (in Japanese).

[5] Matsumoto, T., Amano, Y. and Kiribuchi, Y. (1991). Diagnosis of the types of gallstones by CT. *J. Bil. Tract and Panc.*, **12**, 1205–1212 (in Japanese).

[6] Kuji, T. (1991). Abnormal arrangement of the extra hepatic biliary tract and surgery: The questionnaire survey by the 22nd meeting of the Japanese Study Group on Biliary Surgery, (in Japanese).

[7] Igarashi, Y., Hasegawa, A., Anzai, T. *et al.* (1993). Clinical evaluation of endoscopic lithotomy for stones in the common bile duct. *Endoscopia Digestiva*, **5**, 909–914 (in Japanese).

[8] Hoshi, H., Anzai, T., Ohashi, S. *et al.* (1991). Transpapillary endoscopic treatment for choledocholithiasis; role of peroral choledochoscopy. *Prog. Digest. Endosc.*, **39**, 25–30 (in Japanese).

[9] Maetani, I., Ogawa, S., Hoshi, H. *et al.* (1990). Endoscopic treatment using percutaneus transhepatic cholangioscopy (PTCS). *Prog. Digest. Endosc.*, **36**, 50–55 (in Japanese).

[10] Ohashi, S., Anzai, T., Hoshi, H. *et al.* (1991). Endoscopic treatment for bile duct stone under percutaneous transhepatic cholangioscopy. *Prog. Digest. Endosc.*, **39**, 36–40 (in Japanese).

[11] Tanaka, M., Matsumoto, S., Yoshimoto, H. *et al.* (1985). Fifteen to twenty years' results of surgical sphincterotomy. *Stomach and intestine*, **20**, 1215–1221 (in Japanese).

[12] Umezono, A. and Uematsu, S. (1990). Indication for choledochoduodenostomy. *Surgery*, **52**, 40–44 (in Japanese).

[13] Yokomizo, S., Nakayama, K., Kinoshita, T. *et al.* (1989). Surgical treatment of gallstone diseases and its long-term results. *J. Bil. Tract and Panc.*, **10**, 1221–1226 (in Japanese).

[14] Otani, Y., Tanaka, Y., Goto, K. *et al.* (1993). ESWL: Practical Gastroenterology, 4th Edition. Tokyo Bunkoudo Ltd., 162–167 (in Japanese).

[15] Yamagata, K., Maki, T., Osuga, T. *et al.* (1986). New classification for gallstones in Japan. *J. J. Gastroenterol.*, **83**, 309–316 (in Japanese).

[16] Suzuki, N., Ise, H., Shinya, S. *et al.* (1993). Classification of the cholelithiasis. In: Tsuchiya, H., Matsumoto, Y.: *New trend in treatment for cholelithiasis.* Tokyo Kinbara Ltd., 1–14 (in Japanese).

[17] Maki, T., Matsumoto, T. and Suzuki, N. (1971). Role of sulphated glycoprotein in gallstone formation. *Surg. Gyn. Obst.*, **132**, 846.

[18] Suzuki, N., Ise, H., Shinya, S. *et al.* (1993). Pigmental gall stone. In: Tsuchiya, H. and Matsumoto, Y.: *New trend in treatment for cholelithiasis*, Tokyo Kinbara Ltd., 32–42 (in Japanese).

[19] Kameda, H. (1991). Present status: the types of gallstones by classification in japan. *J. Bil. Tract and Panc.*, **12**, 1179–1183 (in Japanese).

[20] Lemmel, G. (1934). Die Klinische Bedeutung der Duodenal divertikel. Arch fur Verdauungskrankheit, **56**, 59 (in Germany).
[21] Van Hee, R. H. G. G., Van Vooren, W. H. A., Van Hee, W. R. O. P. *et al.* (1979). Vaterian diverticula as a cause of acute pancreatitis. *Acta Hepato Gastroenterol.*, **26**, 170–175.
[22] Culver, Gordon, J. and Pirson, Herbert, S. (1966). The roentgenographic findings in 3 cases of termination of the common bile duct in duodenal diverticula. *Amer. J. Roentg.*, **96**, 370–374.
[23] Yasuda, K., Nakajima, M., Cho, E. *et al.* (1993). Relationship between parapapillary diverticula and biliary tract diseases. *Endoscopia Digestiva*, **5**, 1437–1444 (in Japanese).
[24] Kimura, W., Nagai, H., Kuroda, A. *et al.* (1992). No significant correlation between histologic changes of the papilla of Vater and juxtapapillary diverticulum. *Scand. J. Gastroenterol.*, **27**, 951–956.
[25] Noda, Y., Fujita, N., Kobayashi, G. *et al.* (1993). Parapapillary duodenal diverticulum and endscopic treatment. *Endoscopia Digestiva*, **5**, 1447–1454 (in Japanese).
[26] Fuji, S. and Okita, K. (1990). Common bile duct stones with parapapillary diverticula. *Surgery*, **52**, 8–13 (in Japanese).
[27] Nakamura, H., Kinoshita, H., Hirohashi, K. *et al.* (1994). Suitability of the phrase "accessory hepatic ducts" evaluated from results of direct cholangiography. *J. J. Biliary Association*, **8**, 22–28 (in Japanese).
[28] Hara, H., Isozaki, H., Morita, S. *et al.* (1994). Clinical study of abnormal arrangement of cystic duct. *J. J. Biliary Association*, **8**, 204–208 (in Japanese).
[29] Uchiyama, K., Tanimura, H. and Ishimoto, K. (1993). Types and incidence of abnormal arrangement of the extrahepatic biliary tract assessed by ERCP. Proceedings of the 22nd Study Group of the Japanese Association of Biliary Surgery, 46–47 (in Japanese).
[30] Hoshi, H., Kishi, H., Katagiri, K. *et al.* (1990). The site of junction of the cystic duct and the common bile duct and its configuration in the cases with biriary stones. *Prog. Digest. Endosc.*, **37**, 191–195 (in Japanese).
[31] Kune, G. A. and Sali, A. (1972). *Surgical Anatomy. Current Practice of Biliary Surgery*, 2nd Edition. Boston, Brown and Company Ltd., 1–14.
[32] Ikeda, S., Tanaka, M., Matsumoto, S. *et al.* (1985). Long-term results of endoscopic sphincterotomy. *Stomach and Intestine*, **20**, 1169–1180 (in Japanese).
[33] Tsuyuguchi, T., Saisho, H. and Kurita, S. (1989). Endoscopic papillotomy in the treatment of choledocholithiasis-long term results after small cutting. *J. Bil. Tract and Panc.*, **10**, 1199–1205 (in Japanese).
[34] Hirata, N. and Fujita, R. (1992). Long-term prognosis of endoscopic sphincterotomy. *M. B. Gastro.*, **2**, 35–40 (in Japanese).

